# Dinosolve: a protein disulfide bonding prediction server using context-based features to enhance prediction accuracy

**DOI:** 10.1186/1471-2105-14-S13-S9

**Published:** 2013-10-01

**Authors:** Ashraf Yaseen, Yaohang Li

**Affiliations:** 1 Department of Computer Science, Old Dominion University, Norfolk, VA 23529, USA

## Abstract

**Background:**

Disulfide bonds play an important role in protein folding and structure stability. Accurately predicting disulfide bonds from protein sequences is important for modeling the structural and functional characteristics of many proteins.

**Methods:**

In this work, we introduce an approach of enhancing disulfide bonding prediction accuracy by taking advantage of context-based features. We firstly derive the first-order and second-order mean-force potentials according to the amino acid environment around the cysteine residues from large number of cysteine samples. The mean-force potentials are integrated as context-based scores to estimate the favorability of a cysteine residue in disulfide bonding state as well as a cysteine pair in disulfide bond connectivity. These context-based scores are then incorporated as features together with other sequence and evolutionary information to train neural networks for disulfide bonding state prediction and connectivity prediction.

**Results:**

The 10-fold cross validated accuracy is 90.8% at residue-level and 85.6% at protein-level in classifying an individual cysteine residue as bonded or free, which is around 2% accuracy improvement. The average accuracy for disulfide bonding connectivity prediction is also improved, which yields overall sensitivity of 73.42% and specificity of 91.61%.

**Conclusions:**

Our computational results have shown that the context-based scores are effective features to enhance the prediction accuracies of both disulfide bonding state prediction and connectivity prediction. Our disulfide prediction algorithm is implemented on a web server named "Dinosolve" available at: http://hpcr.cs.odu.edu/dinosolve.

## Background

Disulfide bonds (alternatively called disulfide bridges or SS-bonds) are covalent bonds formed between two sulfur atoms from nonadjacent cysteine pairs of a protein structure. Disulfide bonds are often found in extracellular proteins, which play an important role in folding and enhancing thermodynamic and mechanical stability. Disulfide bonding patterns can also be used to discriminate structure similarity, even when low sequence similarities are present [1]. Furthermore, certain disulfide configurations provide mechanisms for sensing and responding to tensile forces, diversifying and functionalizing protein folds, minimizing aggregation, confining and coupling conformational changes, and controlling packaging and releasing for intercellular transport [2]. Therefore, correctly predicting the formation and connectivity of disulfide bonds can not only reduce the conformational space to aid modeling protein structures in three dimensions, but also help predict important protein functions.

Typically, most of the disulfide bonding prediction approaches involve two stages. The first stage is the bonding state prediction, whose goal is to determine whether each cysteine residue in a protein chain is involved in forming a disulfide bond or not. Afterward, the second stage carries out the connectivity prediction, where cysteine pairs likely to form disulfide bonds are identified.

Since 1990, several methods have been proposed to predict the bonding states of cysteine residues. The early methods used sequence information alone. Muskal et al. [3] implemented a neural network to predict disulfide bonding states and achieved 81% accuracy using a small training set with 689 fragments containing cysteine residues. Fiser et al. [4] proposed a prediction method based on statistical analysis of residue frequencies near the cysteine residues and obtained 71% accuracy on a bigger data set. The main reason that the early methods do not achieve high prediction accuracy is due to the limitation of available protein data set and, more importantly, the restriction to only sequence information. The use of evolutionary information contained in multiple sequence alignments in later disulfide bonding state prediction methods leads to substantial improvements. Fariselli et al. [5] designed a jury of neural networks trained by sequence profiles using multiple sequence alignments and resulted in 81% accuracy. Fiser and Simon [6] derived conservation scores from multiple sequence alignments to predict the oxidation state of cysteine residues and obtained an accuracy of 82%. More recent methods with enhanced strategies and additional features lead to continuing improvements of bonding state prediction accuracy. Mucchielli-Giorgi et al. [7] investigated the contribution of the overall amino acid composition of the protein and managed to increase the accuracy to 84%. Ceroni et al. [8] proposed a method using spectrum kernel in Support Vector Machines (SVMs), which yielded 85% prediction accuracy. Martelli et al. [9] combined a hybrid hidden Markov model and a neural network in their prediction system and reached 84% and 88% accuracy measured on protein basis and cysteine basis, respectively. Song et al. [10] incorporated dipeptide composition as features in prediction and gained similar accuracy.

The pioneered method of connectivity prediction was proposed by Fariselli and Casadio [11] based on graph matching where edges are weighted by residue contact potentials. The reported accuracy is 17 times higher than a random predictor, which is not comparable to the modern predictors with incorporation of evolutionary information in advanced machine learning technologies. Ceroni et al. [12] encoded multiple sequence alignment data into Recursive Neural Networks in their DISULFIND server with 54.5% pattern precision and 60.2% bonded pair accuracy. Ferre and Clote [13] took advantage of secondary structure encoding in their DiANNA server and reached 86% accuracy (for both bonded and non-bonded). Cheng et al. [14] performed large-scale prediction of disulfide connectivity using kernel methods, two-dimensional recursive neural networks, and weighted graph matching and obtained accuracy of 51% pattern precision. Vincent et al. [15] took advantage of decomposition kernels for classifying chains instead of individual residues and achieved prediction accuracy comparable to the other prediction methods.

Computational approaches toward the prediction of disulfide bonding states and disulfide connectivity pattern are mostly machine learning approaches, including statistical analysis, neural networks, SVM, hidden Markov Chains, etc. Features influencing the formation of disulfide bonds, such as multiple sequence alignment, secondary structures, number of cysteine residues in a protein chain, etc., are encoded in the machine learning algorithms to improve prediction accuracy. Therefore, extracting and selecting "good" features are critical to the performance of the learning machines.

In the very beginning methods of predicting disulfide bonding states, the training set contains only 689 samples with cysteine residues [3]. As of August 21, 2012, the Protein Data Bank (PDB) includes 83,983 protein structure entries. The protein data sets Cull7987 (25% sequence identity, 3.0A resolution, and 1.0 R-factor cutoff) and Cull16633 (50% sequence identity, 3.0A resolution, and 1.0 R-factor cutoff) generated by the PISCES server [16] contains 22,475 and 51,990 cysteine residues, respectively. These available protein structures provide rich information resource to extract advanced statistical features for further improvement of disulfide bonding state and connectivity prediction accuracies.

In this paper, we investigate the approaches of deriving context-based scores based on the mean-force potentials derived from a large cysteine sample set. We consider not only the first-order interactions, but also the second-order interactions. Because of the recently increasing number of experimentally determined protein structures in PDB, we have sufficient number of samples to efficiently estimate the second-order mean-force potentials. Afterward, context-based scores for cysteine residues considering nearby neighbors at different distances are generated. These context-based scores are then incorporated as features together with the multiple sequence alignment data to train neural networks for disulfide bonding state and connectivity prediction. 10-fold cross validations are performed. We also test our method on several commonly used protein benchmarks, including Manesh215, Carugo338, and CASP9 targets.

## Methods

### The protein data sets

We use the protein chain dataset Cull16633 generated by the PISCES server [16] on 10/21/2011 to collect cysteine samples to generate context-based statistics and for neural network training as well. Cull16633 contains 16,633 chains with at most 50% sequence identity, 3.0A resolution cutoff, and 1.0 R-factor. Chains without cysteine residues or with only one cysteine residue are eliminated. We also eliminate very short chains whose lengths are less than 40 residues since the PSI-BLAST program [17] is usually unable to generate profiles for very short sequences. The disulfide bond assignments are determined by the DSSP program [18]. Inter-chain disulfide bonded cysteines are excluded from the data set as well. Moreover, cysteine residues with undetermined structures are excluded.

After eliminating all unfavorable chains, the total number of protein chains containing at least two cysteine residues remained in Cull16633 is 9,781. We refer to this protein chain set as Cull50. The total number of cysteine residues in Cull50 is 47,655. 21.27% of these cysteine residues are bonded. We also use another dataset Cull7986 generated from PISCES server with maximum 25% sequence identity, 3.0A resolution, and 1.0 R-factor. After filtering, the total number of protein chains containing at least two cysteine residues is 4,340 with a total of 20,309 cysteine residues, where 21.28% of those are bonded. This protein chain set is referred to as Cull25. We compare the performance of our prediction methods when Cull50 and Cull25 are used as training sets.

The recent CASP9 targets [19] as well as the public protein data sets Manesh215 [20] and Carugo338 [21], which are popularly employed as benchmarks for secondary structure predictions, are used to benchmark our method. Therefore, any sequences with greater than 25% similarity with the test benchmarks sequences are excluded from the Cull50 and Cull25 when the neural networks are trained and also when the context-based scores are generated.

### Context-based statistics

**Figure 1:**
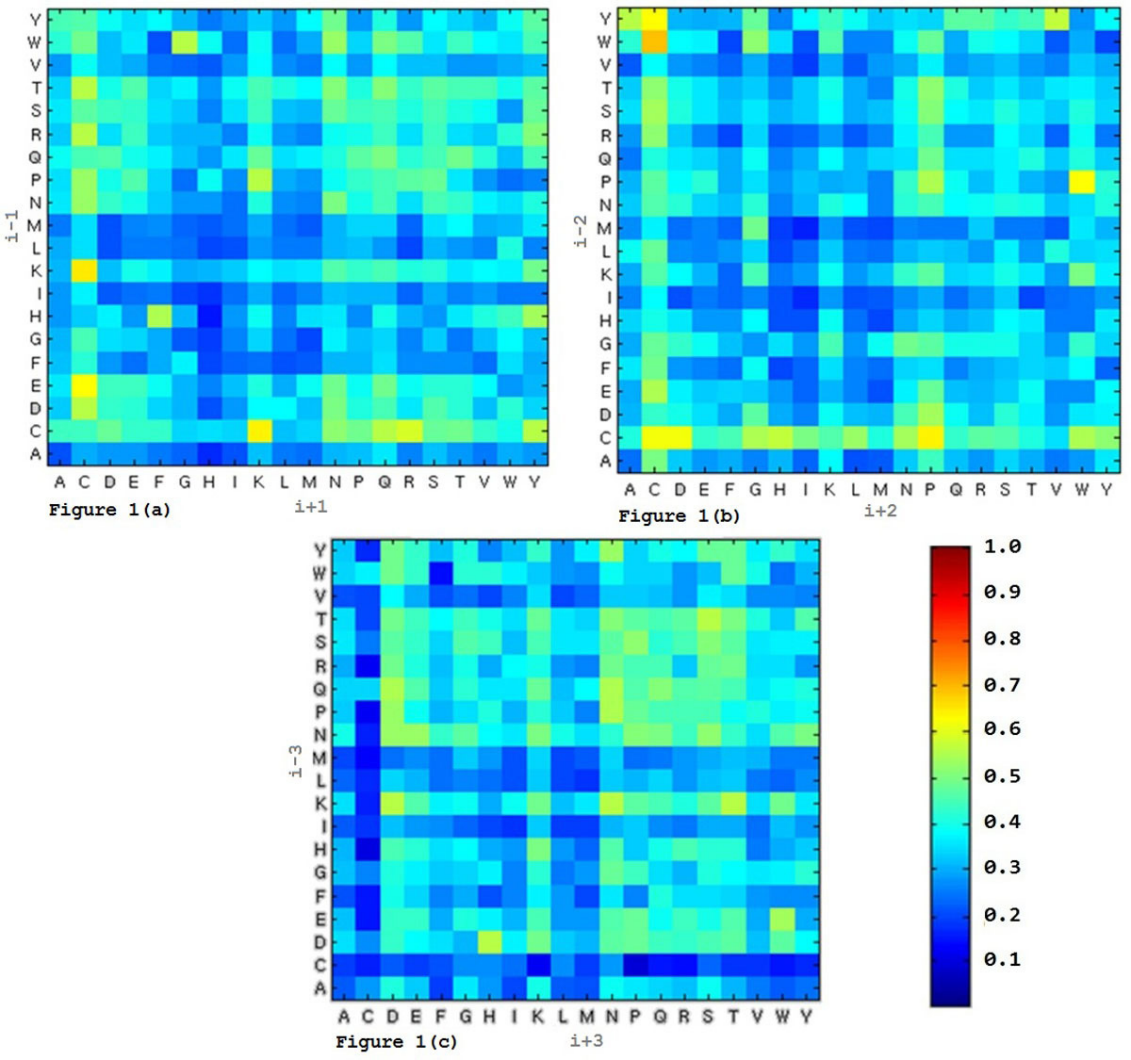
**Probability of cysteine in disulfide bonding state with neighbors at different positions**. (a) Probability of cysteine in bonding state with neighbors at i - 1 and i + 1 positions (b) Probability of cysteine in bonding state with neighbors at i - 2 and i + 2 positions (c) Probability of cysteine in bonding state with neighbors at i - 3 and i + 3 positions

It is well known that there exist general short range regularities in the primary structure of proteins [22]. Presumably, the neighboring residues have strong and probably deterministic influence to the chemical property of cysteine in forming disulfide bond [3]. Actually, cysteine often forms particular motifs of biochemical functions with neighboring residues, such as Cys-X-X-Ser [23], Cys-X-X-Cys [24], Leu-X-Cys-X-Glu [25], Cys-X-X-Asp-X-X-Cys [26], etc. Figures 1(a), 1(b), and 1(c) show the probability of cysteine at position *i *in disulfide bonding state with the neighboring residues at *i *- 1 and *i *+ 1, *i *- 2 and *i *+ 2, and *i *- 3 and *i *+ 3 positions, respectively. One can notice that the neighboring residues separated by two residues in the middle still have strong influences on the bonding state of the center cysteine residue.

**Figure 2:**
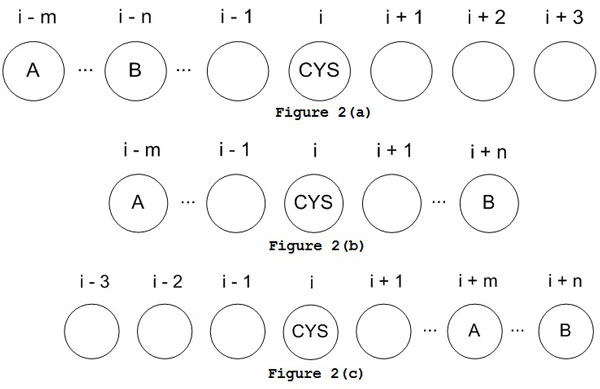
**Three possible positions of two neighbors to a cysteine residue**. (a) Both neighbors on the left hand side of cysteine (b) Two neighbors on both sides of cysteine (c) Both neighbors on the right hand side of cy steine

In this work, we derive the mean-force potentials [27] to estimate the favorability of a cysteine residue in a bonding state within its amino acid environment. The mean-force potential is based on the derived statistics of correlations between the cysteine residue and its nearby neighbors. In particular, the increasing number of experimentally determined protein structures in PDB recently has provided sufficient number of samples to enable derivation statistics for second-order mean-force potential. In our method, the first-order statistics estimate the correlations between a cysteine residue and one of its neighboring residues while the second-order statistics estimate the correlations between a cysteine residue and the coexistence of two neighboring residues. Both first-order and second-order statistics are extracted from protein chains in the Cull datasets. For a cysteine sample with window size of *K*, there are *K *- 1 position combinations for first-order statistics in total. Figure 2 shows the three possible situations of two neighbors relative to a cysteine residue when extracting second-order statistics, including (a) both neighbors on the left; (b) two neighbors on both sides; and (c) both neighbors on the right. Therefore, considering a window size of *K *for a cysteine sample, there are totally $\left(\begin{array}{c} \text{K}-1\\ 2 \end{array}\right)$ position combinations for the second-order statistics of a cysteine residue in bonding state.

**Figure 3:**
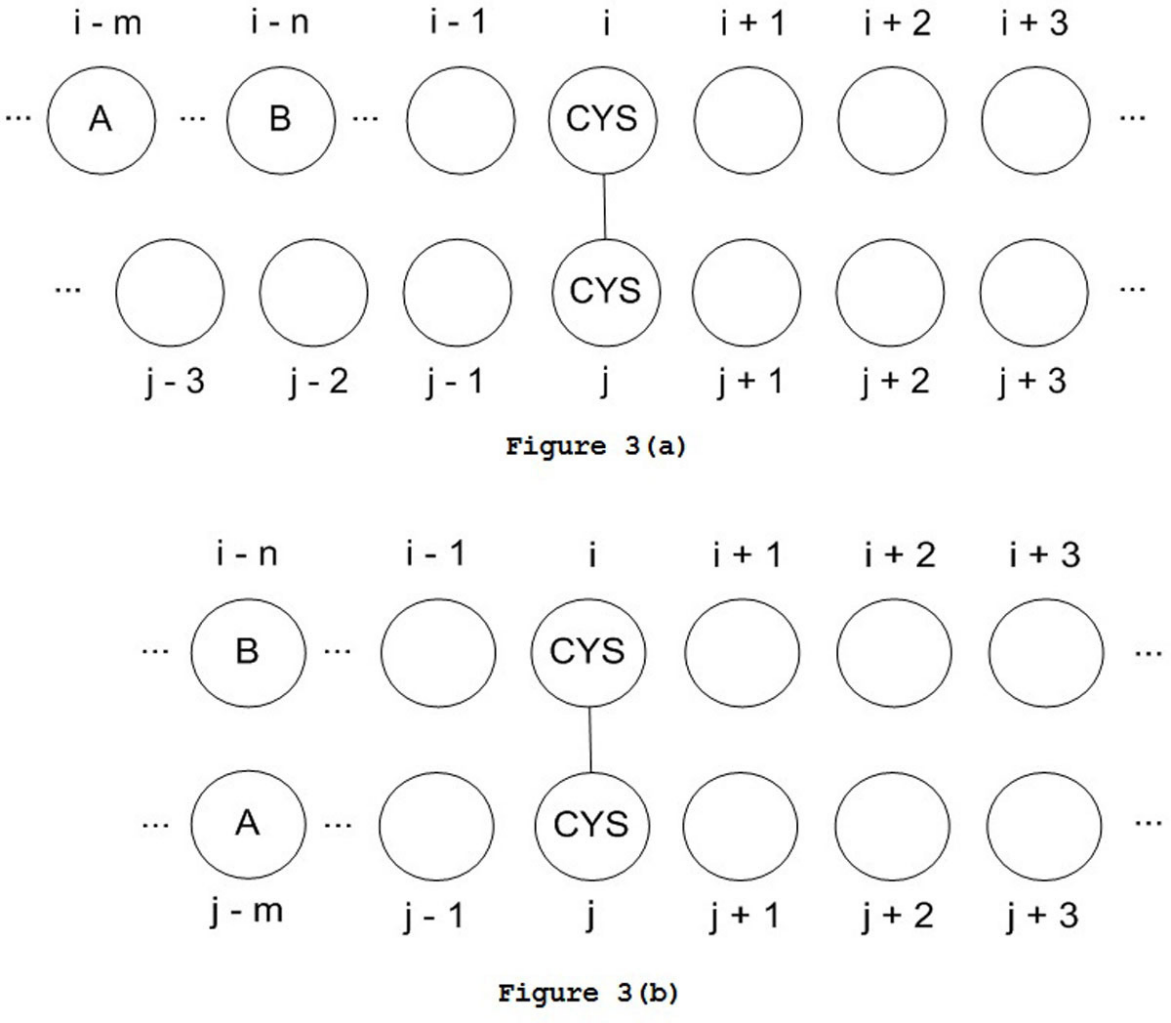
**Possible positions of two neighbors to a cysteine residue pair in disulfide bond**. (a) Both residues are the neighbors of one cysteine residue (b) One residue is the neighbor of cysteine i and the other is the neighbor of cysteine j

Similar to the bonding state statistics, the first-order and second-order statistics of a disulfide bonded cysteine pair related to its neighboring residues are also extracted from the PDB. These statistics are used to estimate the probability of a cysteine pair in forming disulfide connectivity. Compared to the statistics in estimating a cysteine residue in a bonding state, the main difference lies in the different number of position combinations in second-order statistics since the two neighboring residues may belong to two different cysteine residues. Figure 3(a) shows the situation that both neighboring residues belong to one cysteine residue and Figure 3(b) shows the situation that the two neighboring residues belong to different cysteine residues. Therefore, considering a window size of *K *for both cysteine residues connected in a disulfide bond, there are totally $\left(\begin{array}{c} 2K-2\\ 2 \end{array}\right)/2$ position combinations for the second-order statistics of a bonding cysteine pair.

To obtain more precise neighboring correlation statistics to disulfide bonding states, we consider the divergence of a protein sequence in its structural family by using the Position Specific Scoring Matrix (PSSM) data specifying the frequency of each amino acid type in a protein multiple sequence alignment. The PSSM data is generated by running the PSI-BLAST program with three iterations (E value: 0.001) of searching against the non-redundant database of protein sequences (NR). Let *R_i _*denote residue *R *at position *i *in a protein sequence and let *R_(j) _*denote residue *R *at relative position *j *to a cysteine residue. In the first-order statistics, the observed probability, $P_{obs}\left(Bonded|R_{\left(k\right)}\right)$, of residue type *R *with relative distance *k *to a bonded cysteine in a specific protein data set is estimated as

$$P_{obs}\left(Bonded|R_{\left(k\right)}\right)=\frac{\sum_{protein}\sum_{CYS_{i}\;is\;bonded}PSSM\left(R_{i+k}\right)*PSSM\left(CYS_{i}\right)}{\sum_{protein}\sum_{CYS_{i}\;is\;bonded}PSSM\left(CYS_{i}\right)},$$

where $PSSM\left(R_{i}\right)$ is the PSSM frequency of residue type *R *at position *i *in a protein sequence. Similarly, in the second-order statistics, the observed probability, $P_{obs}\left(Bonded|R_{\left(k_{1}\right)},R_{\left(k_{2}\right)}\right)$, of the coexistence of residues $R_{\left(k_{1}\right)}$ and $R_{\left(k_{2}\right)}$ to a bonded cysteine is estimated as

$$P_{obs}\left(Bonded|R_{\left(k_{1}\right)},R_{\left(k_{2}\right)}\right)=\frac{\sum_{protein}\sum_{CYS_{i}\;is\;bonded}PSSM\left(R_{i+k_{1}}\right)*PSSM\left(CYS_{i}\right)*PSSM\left(R_{i+k_{2}}\right)}{\sum_{protein}\sum_{CYS_{i}\;is\;bonded}PSSM\left(CYS_{i}\right)}.$$

The neighboring correlation statistics to the disulfide bonding pair are obtained in a similar manner.

### Context-based potential

The context-based potential for cysteine bonding state is generated based on the potentials of mean force method [27]. In this work, we consider the first-order and the second-order mean-force potentials only. Currently, there is insufficient number of available protein structures in PDB to derive meaningful statistics for estimating higher order interactions.

According to the inverse-Boltzmann theorem, we introduce the first-order mean-force potential $U\left(R_{\left(k\right)},Bonded\right)$ to treat the interaction between residue $R_{\left(k\right)}$ and cysteine in forming a disulfide bond,

$$U\left(R_{\left(k\right)},Bonded\right)=-RTln\frac{P_{obs}\left(Bonded|R_{\left(k\right)}\right)}{P_{ref}\left(Bonded|R_{\left(k\right)}\right)}$$

Here *R *is the gas constant, *T *is the temperature, and $P_{ref}\left(Bonded|R_{\left(k\right)}\right)$is the reference state, which is estimated as

$$P_{ref}\left(Bonded|R_{\left(k\right)}\right)=\frac{\sum_{protein}\sum_{CYS_{i}}PSSM\left(R_{i+k}\right)*PSSM(CYS_{i})}{\sum_{protein}\sum_{CYS_{i}}PSSM(CYS_{i})}.$$

Similarly, the second-order mean-force potential $U\left(R_{\left(k_{1}\right)},R_{\left(k_{2}\right)},Bonded\right)$ is calculated as

$$U\left(R_{\left(k_{1}\right)},R_{\left(k_{2}\right)},Bonded\right)=-RTln\frac{P_{obs}\left(Bonded|R_{\left(k_{1}\right)},R_{\left(k_{2}\right)}\right)P_{ref}\left(Bonded|R_{\left(k_{1}\right)}\right)P_{ref}\left(Bonded|R_{\left(k_{1}\right)}\right)}{P_{ref}\left(Bonded|R_{\left(k_{1}\right)},R_{\left(k_{2}\right)}\right)P_{obs}\left(Bonded|R_{\left(k_{1}\right)}\right)P_{obs}\left(Bonded|R_{\left(k_{1}\right)}\right)}$$

with the second-order reference state,

$$P_{ref}\left(Bonded|R_{\left(k_{1}\right)},R_{\left(k_{2}\right)}\right)=\frac{\sum_{protein}\sum_{CYS_{i}}PSSM\left(R_{i+k_{1}}\right)*PSSM\left(CYS_{i}\right)*PSSM\left(R_{i+k_{2}}\right)}{\sum_{protein}\sum_{CYS_{i}}PSSM(CYS_{i})}.$$

Influenced by all of its neighboring residues, the overall mean-force potential for the interactions of a cysteine residue in bonding state is the summation of all first-order and second-order potentials while the higher-order interactions are ignored

$$U(CYS_{i},\;Bonded)=\sum_{k}^{k\neq 0}U\left(R_{\left(k\right)},Bonded\right)+\sum_{k_{1}}^{k_{1}\neq 0}\sum_{k_{2}}^{k_{2}\neq 0}U\left(R_{\left(k_{1}\right)},R_{\left(k_{2}\right)},Bonded\right).$$

The potential $U\left(CYS_{i},CYS_{j},Connected\right)$ for a bonded cysteine pair $CYS_{i}$ and $CYS_{j}$ can be obtained in a similar way. These potentials are used as context-based scores to be encoded in neural network training for bonding state and connectivity predictions.

### Neural network model

We adopt the standard feed-forward back-propagation neural network architecture for both disulfide bonding state prediction and connectivity prediction. The neural networks contain a single hidden layer, an input layer, and an output layer.

**Figure 4:**
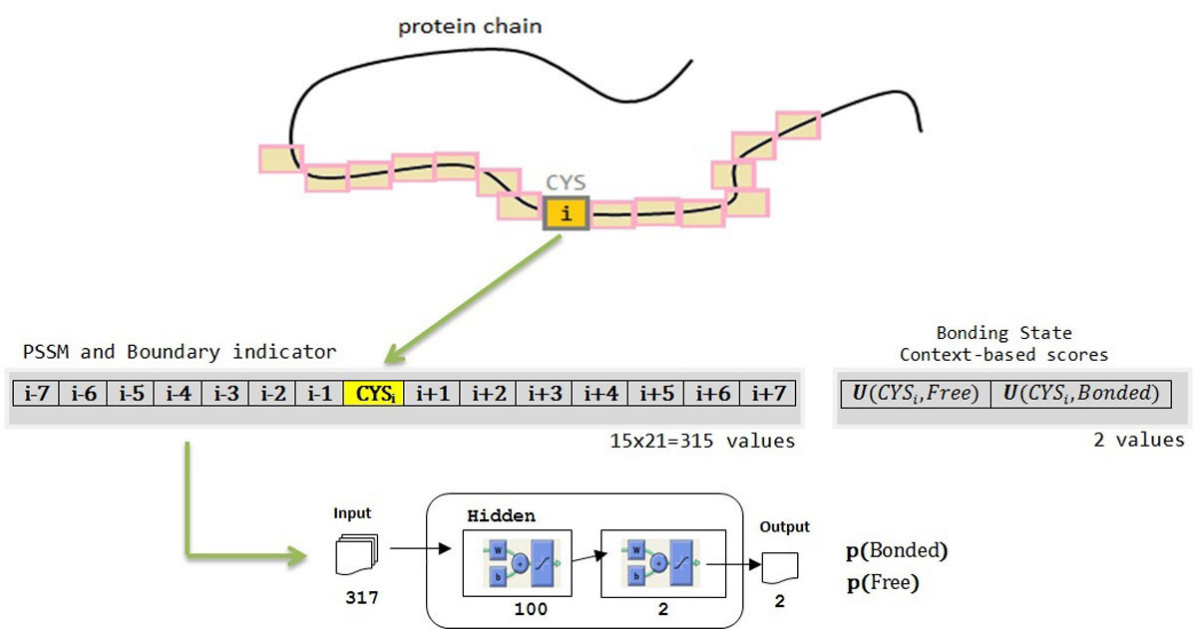
Encoding and neural network architecture for disulfide bonding state prediction

The neural network for bonding state prediction uses a window size of 15 residues for input encodings. Each residue is represented with 20 values from the PSSM data and 1 extra input to indicate if the window overlaps C-terminal or N-terminal. When incorporating the context-based scores in training the neural network predictor, two more inputs specifying the scores of the cysteine residue being in free and bonding state are added. Hence, a total number of 317 values are used to describe each cysteine residue. 100 hidden nodes are used in the neural network for bonding state prediction. Figure 4 depicts the encoding and neural network architecture for disulfide bonding state prediction.

**Figure 5:**
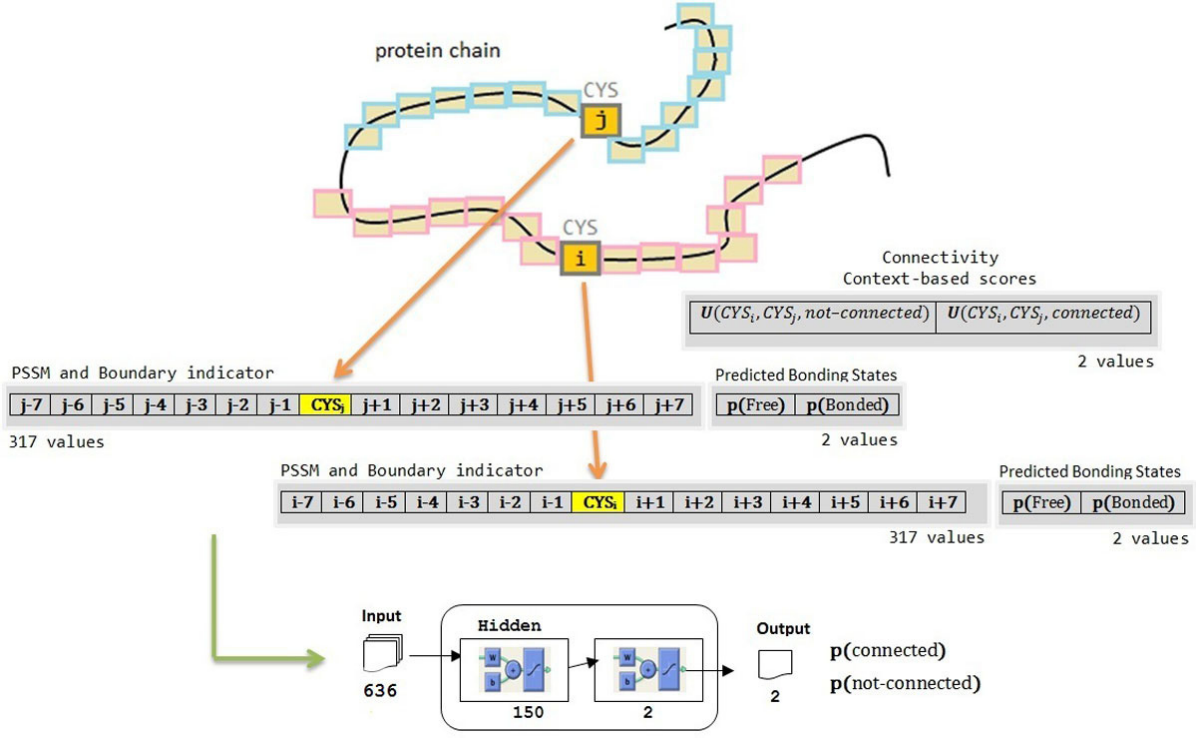
Encoding and neural network architecture for disulfide connectivity prediction

The neural network for connectivity prediction incorporates two windows, each with size of 15 residues, for input encoding. Each window encodes the amino acid environment of a cysteine residue in a cysteine pair. Each residue is encoded with 20 PSSM values and 1 boundary indicator. The predicted results (bonded or free) from the bonding state prediction for both cysteine residues and the context-based scores for connectivity are also encoded as input. As a result, there are totally 636 input values for each cysteine pair. 150 hidden nodes are used in the neural network for connectivity prediction. Figure 5 illustrates the encoding and neural network architecture for disulfide connectivity prediction.

### Performance measures

We use sensitivity (*Sn*), specificity (*Sp*), and Matthew's correlation coefficient (*Mcc*) to measure the quality of our cysteine bonding state and connectivity prediction. The definitions of sensitivity (*Sn*), specificity (*Sp*), and Matthew's correlation coefficient (*Mcc*) are given by,

Sn=TN(TP+FN)

Sp=TN(TN+FP)

$$Mcc=\frac{\left(TP*TN-FN*FP\right)}{\sqrt{\left(TP+FN\right)*\left(TN+FP\right)*\left(TP+FP\right)*\left(TN+FN\right)}},$$

where *TP*, *TN*, *FP*, and *FN *are the number of true positives, the number of true negatives, the number of false positives, and the number of false negatives, respectively. We also use residue-level accuracy ($Q_{c}$) and protein-level accuracy ($Q_{p}$) to measure the prediction accuracy. The residue-level accuracy $Q_{c}$ is defined as

Qc=PcNc,

where $P_{c}$ is the total number of correctly predicted cysteine residues and $N_{c}$ is the total number of cysteine residues. The protein-level accuracy $Q_{p}$ is defined as

Qp=PpNp,

where $P_{p}$ is the total number of proteins where the bonding states of all of its cysteine residues are correctly predicted and $N_{p}$ is the total number of proteins in the data set.

### *N*-fold cross validation

To have a reliable estimation of the prediction accuracy, we employ the *N*-fold cross validation approach on the Cull data sets. The protein chains in the cull data sets are divided into *N *subsets with approximately the equal size. At each step, *N *- 2 subsets are used for neural network training while the other 2 are used separately for testing and validation. The process is repeated *N *times. The overall prediction accuracy is calculated as the average of the accuracies of the *N *folds.

## Results

### The bonding state prediction

Table 1 compares the prediction qualities of bonding states with PSSM-only encoding and PSSM with context-based scores encoding after 10-fold cross validation. Compared to the one trained with PSSM data only, the neural network using context-based scores as additional features results in improvements in all performance indexes, including *S_n_*, *S_p_*, *Q_c_*, *Q_p_*, and *Mcc*. The residue-level prediction accuracy (0.908) and protein-level prediction accuracy (0.856) are higher than the reported accuracies in [3-15]. Table 1 also compares the prediction qualities when Cull25 and Cull50 are used as training sets. Cull50 has more than twice cysteine samples as Cull25, which leads to better prediction performance than Cull25.

**Table 1:** Comparison of prediction performance of bonding states using PSSM only and PSSM with context-based scores on Cull25 and Cull50 using 10-fold cross validation

	Cull25		Cull50	
	**PSSM Only**	**PSSM+Score**	**PSSM Only**	**PSSM+Score**

*S_n_*	0.554	0.616	0.655	0.720

*S_p_*	0.945	0.956	0.947	0.959

*Q_c_*	0.870	0.888	0.885	0.908

*Q_p_*	0.719	0.751	0.829	0.856

*Mcc*	0.574	0.646	0.734	0.801

### Connectivity prediction

Table 2 compares the computational results of 10-fold cross validation for disulfide bond connectivity predictions on Cull50 using PSSM-only and PSSM with context-based scores for neural network encoding. Similar to bonding state prediction, one can find that incorporating the context-based scores as features in neural network training enhances the connectivity prediction accuracy, where sensitivity (*Sn*), specificity (*Sp*), and overall accuracy (*Q_c_*) are improved from 73.07%, 91.03%, and 86.91% to 73.42%, 91.61%, and 87.34%, respectively, compared to PSSM only encoding. These prediction results are also higher than the reported disulfide connectivity accuracies in the popular disulfide bond prediction servers [11-15].

**Table 2:** Computational results of 10-fold cross validation on Cull50 using PSSM only and PSSM + Score in neural network encoding

	PSSM only			PSSM + Score		
**fold**	**Sn**	**Sp**	**Qc**	**Sn**	**Sp**	**Qc**

1	73.90	91.60	87.50	74.90	91.60	87.70

2	72.80	93.00	88.10	71.70	93.10	88.00

3	70.70	91.90	86.50	71.40	92.40	87.10

4	78.80	82.30	82.20	77.80	84.10	82.60

5	75.20	91.40	87.60	74.10	92.00	87.80

6	71.40	92.30	87.70	71.30	93.00	88.10

7	74.50	92.40	88.50	76.00	92.40	88.80

8	66.80	93.60	87.40	70.40	93.30	88.00

9	69.00	90.20	85.20	68.40	91.50	86.10

10	77.60	91.60	88.40	78.20	92.70	89.20

Average	73.07	91.03	86.91	73.42	91.61	87.34

Table 3 lists the prediction results on chains in Manesh215, Carugo338, and CASP9, which include at least one disulfide bond. The percentage of chains where all disulfide bonds are correctly predicted is 87.8%.

**Table 3:** Prediction performance on protein chains in Manesh215, Carugo338, and CASP9

	Manesh215		Carugo338		CASP9		All	
**# of disulfide bonds**	**# of chains**	**# of correctly predicted**	**# of chains**	**# of correctly predicted**	**# of chains**	**# of correctly predicted**	**# of chains**	**# of correctly predicted**

1	14	13	23	23	1	1	38	37

2	12	11	21	21	0	0	33	32

3	9	7	19	16	1	1	29	24

4	3	2	13	12	0	0	16	14

5	3	3	6	5	0	0	9	8

6	1	0	2	2	0	0	3	2

7	1	1	2	1	0	0	3	2

8	2	1	2	2	0	0	4	3

9	0	0	3	0	0	0	3	0

10	0	0	0	0	0	0	0	0

11	0	0	0	0	0	0	0	0

12	0	0	0	0	0	0	0	0

13	0	0	0	0	1	0	1	0

Summary						139	122 (87.8%)	

**Figure 6:**
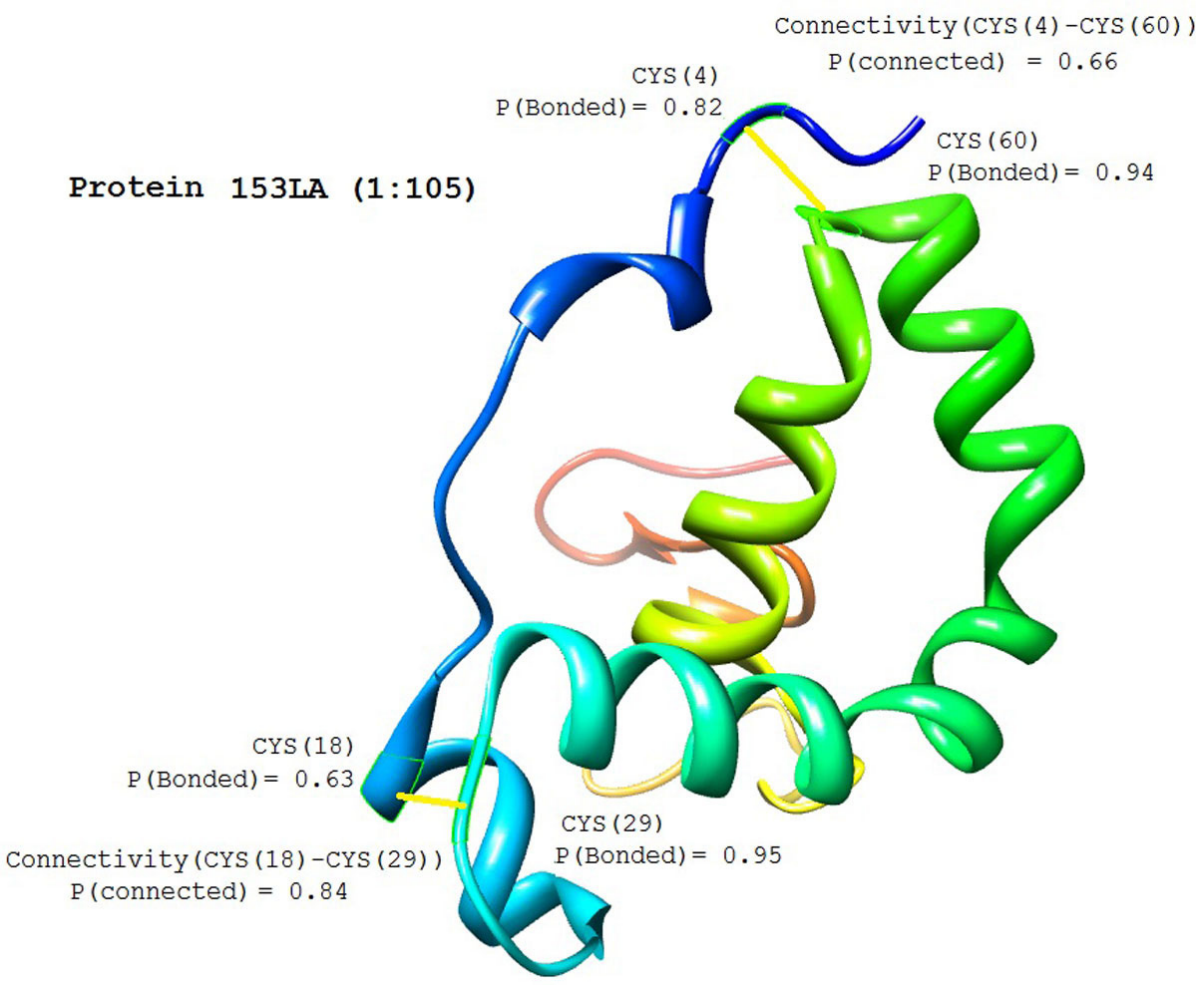
Disulfide connectivity prediction on protein 153L A chain

**Figure 7:**
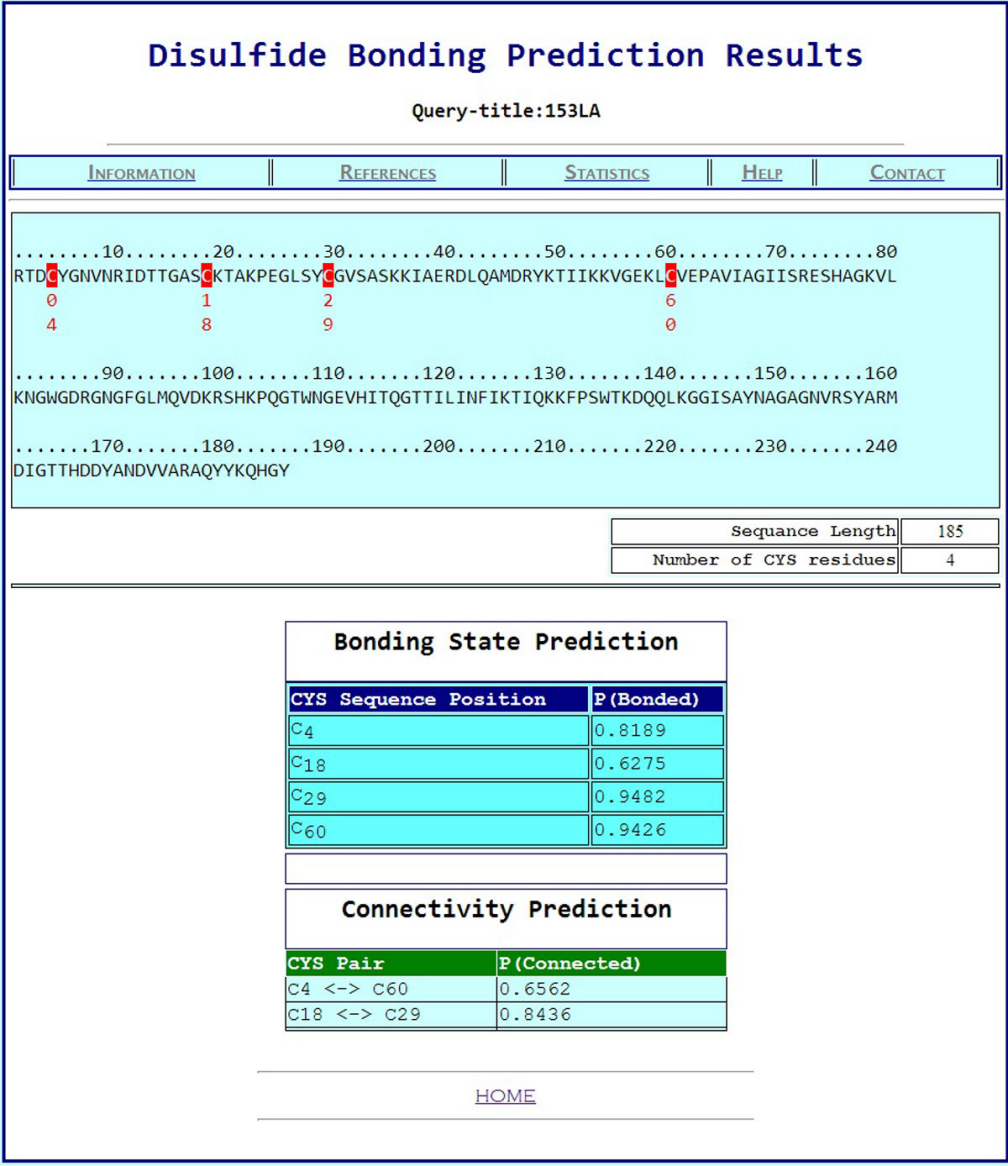
A snapshot of the prediction result after submitting a query sequence (of protein chain 153LA) using our disulfide prediction webserver (dinosolve)

Figure 6 depicts an example of the disulfide connectivity prediction on protein 153L chain 'A' listed in Manesh215. The native 153L(A) structure has four cysteine residues: CYS(4), CYS(18), CYS(29), and CYS(60). CYS(4) is connected to CYS(60) and CYS(29) is connected to CYS(60) by disulfide bonds. In the bonding state prediction, the predicted bonding probabilities for CYS(4), CYS(18), CYS(29), and CYS(60) are 0.82, 0.84, 0.95, and 0.94, respectively, which are all higher than 0.5 indicating that they are all bonded. In the connectivity prediction, the predicted bonding probabilities for the potential disulfide bonds are listed in Table 4. From Table 4, one can find that CYS(18) and CYS(60) are most likely to be connected due to their highest predicted connectivity probability (0.90). However, if CYS(18) and CYS(60) are connected, CYS(4) and CYS(29) are unlikely to be connected due to their low predicted connectivity probability (0.32), which violates the predicted results during bonding state prediction. Therefore, an alternative connectivity pattern is selected with CYS(18)-CYS(29) and CYS(4)-CYS(60). This prediction result matches the disulfide connectivity pattern in the native structure of 153L(A). Figure 7 shows a snapshot from our web-based disulfide bonding prediction server (Dinosolve) for the prediction results of protein chain 153L(A).

**Table 4:** Predicted bonding probability for potential disulfide bonds in 153L(A)

Potential Disulfide Bonds	Predicted Bonding Probability
CYS(4)-CYS(18)	0.37

CYS(4)-CYS(29)	0.32

CYS(4)-CYS(60)	0.66

CYS(18)-CYS(29)	0.84

CYS(18)-CYS(60)	0.90

CYS(29)-CYS(60)	0.34

## Discussion

The context-based scores are effective features to enhance the neural network training process. When context-based scores are incorporated, the bonding state prediction accuracies are improved on all three benchmarks compared to those using PSSM data only. Table 5 shows the comparison of residue-level accuracies ($Q_{c}$) on the popularly used public benchmarks, including Manesh215, Carugo338, and CASP9. Similar to the computational results of 10-fold cross validation, one can find that the Cull50 training set yields better prediction performance than Cull25.

**Table 5:** Comparison of residue-level accuracies (Qc) on benchmarks of Manesh215, Carugo338, and CASP9 using Cull25 and Cull50 as training sets

	Cull25		Cull50	
	**PSSM Only**	**PSSM+Score**	**PSSM Only**	**PSSM+Score**

Manesh215	0.830	0.848	0.879	0.900

Carugo338	0.808	0.821	0.872	0.884

CASP9	0.950	0.951	0.955	0.963

Moreover, incorporating the context-based scores as features in neural network training enhances the connectivity prediction accuracy, where sensitivity (*S_n_*), specificity (*S_p_*), and overall accuracy (*Q_c_*) are improved from 73.07%, 91.03%, and 86.91% to 73.42%, 91.61%, and 87.34%, respectively, compared to PSSM only encoding.

**Figure 8:**
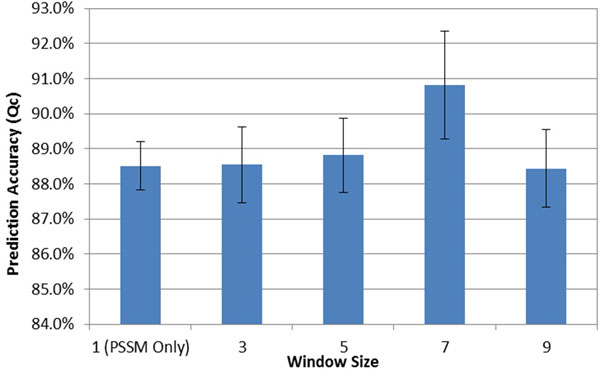
10-fold cross validated accuracies using context-based scores generated with different window sizes

One important question for generating the context-based statistics is how faraway the neighbors in sequence need to be involved. Figure 8 compares the 10-fold cross validated accuracies when context-based features with different window sizes are used for neural network training. One can find that the context-based features with window sizes 3 and 5 slightly improve the prediction accuracy compared to using PSSM only. However, the context-based features with window size 7 yield the optimal performance. This is mainly due to the fact that the context-based features with window size 7 take the important *i *- *i*+3 residue correlations into account, where such correlations are often found in many motifs where cysteine is involved, such as Cys-X-X-Cys, Cys-X-X-Ser, Cys-X-X-His, Cys-X-X-Pro, Cys-X-X-Asp, etc. Another reason is, when the window size 7 is used, the residue-residue correlations in secondary structures are implicitly estimated, because helices, strands, and coils are strongly correlated at relative positions *i*-3 - *i *- *i*+3, *i*-2 - *i *- *i*+2, and *i*-1 - *i *- *i*+1, respectively [28]. It is also interesting to find that the prediction accuracy drops when the context-based features with window size 9 are employed. This is because the context-based scores with window size 9 integrate almost twice as many mean-force potential terms as scores with window size 7 - these additional terms measure the long distance inter-residue correlations of *i *- *i*+4, which are not as important as the shorter inter-residue correlations but accumulate the statistical sampling noise.

## Conclusions

An approach of deriving context-based scores based on the mean-force potentials for characterizing the favorability of cysteine residues in disulfide bond according to their amino acid environment is developed in this paper. Recently, the increasing number of experimentally determined protein structures in PDB has made sufficient number of cysteine samples available. This enables us to obtain reliable statistics for second-order mean-force potentials and thus leads to context-based scores with better accuracy. These context-based scores are selected as features together with other sequence and evolutionary information in neural network training for disulfide bonding state and connectivity predictions. The effectiveness of using context-based features has been demonstrated in our computational results in 10-fold cross validation as well as on benchmarks of Manesh215, Carugo338, and CASP9, where enhancements of prediction accuracies in both bonding state and connectivity predictions are observed. In disulfide bonding state prediction, our 10-fold cross validated accuracy is 90.8% at residue-level and 85.6% at protein-level, which is around 2% improvement compared to the best reported results [3-10] in bonding state prediction, to the best of our knowledge. In disulfide bonding connectivity prediction, our method yields overall sensitivity of 73.42% and specificity of 91.61%, which are also higher than the reported disulfide connectivity accuracies in the popular disulfide bond prediction servers [11-15].

Although the improvement of our method is relatively small (~2% accuracy improvement), our 10-fold cross validated accuracy has reached 90% at amino acid level for bonding state prediction, which is rather high accuracy compared to many other computational protein structure modeling problems. Also, from tertiary structure prediction point of view, reducing inaccuracy, even just a few percent, would be very helpful in modeling efficiency, because the search space for finding a tertiary structure goes up superlinearly with the fraction of inaccuracy. Furthermore, our method of generating context-based statistics relies on the number of cysteine residues presented in known protein structures in PDB. As the number of protein crystal structures available in PDB continues to increase rapidly, we will be able to obtain more accurate context-based statistics for disulfide bonding and thus our method has potential to achieve further accuracy improvement in the future.

A web server called "Dinosolve" implementing our disulfide bonding prediction method is available at http://hpcr.cs.odu.edu/dinosolve. Services of both bonding state and connectivity predictions are provided.

## Competing interests

The authors declare that they have no competing interests.

## Authors' contributions

YL conceived the context-based scoring method. AY implemented the method and carried out the computation. AY and YL performed the result analysis. Both authors read and approved the final manuscript.

## Declarations

This work is supported by NSF grant 1066471 and ODU 2013 Multidisciplinary Seed grant.

This article has been published as part of BMC Bioinformatics Volume 14 Supplement 13, 2013: Selected articles from the 9th Annual Biotechnology and Bioinformatics Symposium (BIOT 2012). The full contents of the supplement are available online at http://www.biomedcentral.com/bmcbioinformatics/supplements/14/S13

## References

[B1] Chuang C Chen C Yang J Lyu P Hwang J Relationship between protein structures and disulfide-bonding patterns Proteins 2003 14 1 1 5 12945044 10.1002/prot.10492

[B2] Fass D Disulfide Bonding in Protein Biophysics Annu Rev Biophys 2012 14 63 79 22224600 10.1146/annurev-biophys-050511-102321

[B3] Muskal S Holbrook S Kim S Prediction of the disulfide-bonding state of cysteine in proteins Protein Engineering 1990 14 8 667 672 2217140 10.1093/protein/3.8.667

[B4] Fiser A Cserzo M Tudos E Simon I Different sequence environments of cysteines and half cystines in proteins: Application to predict disulfide forming residues FEBS Letters 1992 14 117 120 1633841 10.1016/0014-5793(92)80419-h

[B5] Fariselli P Riccobelli P Casadio R Role of evolutionary information in predicting the disulfide-bonding state of cysteine in proteins Proteins: Structure, Function, and Genetics 1999 14 340 346 10409827

[B6] Fiser A Simon I Predicting the oxidation state of cysteines by multiple sequence alignment Bioinformatics 2000 14 3 251 256 10869018 10.1093/bioinformatics/16.3.251

[B7] Mucchielli-Giorgi M Hazout S Tuffery P Predicting the disulfide bonding state of cysteines using protein descriptors Proteins: Structure, Function, and Bioinformatics 2002 14 3 243 249 10.1002/prot.10047 11835499

[B8] Ceroni A Frasconi P Passerini A Vullo A Predicting the disulfide bonding state of cysteines with combination of kernel machines J VLSI Signal Processing 2003 14 287 295

[B9] Martelli P Fariselli P Malaguti L Casadio R Prediction of the disulfide bonding state of cysteines in proteins with hidden neural networks Protein Engineering 2002 14 12 951 953 12601133 10.1093/protein/15.12.951

[B10] Song J Wang M Li W Xu W Prediction of the disulfide-bonding state of cysteines in proteins based on dipeptide composition Biochemical and Biophysical Research Communications 2004 14 1 142 147 15110765 10.1016/j.bbrc.2004.03.189

[B11] Fariselli P Casadio R Prediction of disulfide connectivity in proteins Bioinformatics 2001 14 10 957 964 11673241 10.1093/bioinformatics/17.10.957

[B12] Ceroni A Passerini A Vullo A Frasconi P DISULFIND: a disulfide bonding state and cysteine connectivity prediction server Nucleic Acids Research 2006 14 W177 W181 16844986 10.1093/nar/gkl266 PMC1538823

[B13] Ferre F Clote P DiANNA: a web server for disulfide connectivity prediction Nucleic Acids Research 2005 14 W230 W232 15980459 10.1093/nar/gki412 PMC1160173

[B14] Cheng J Saigo H Baldi P Large-scale prediction of disulphide bridges using kernel methods, two-dimensional recursive neural networks, and weighted graph matching Proteins: Structure, Function, and Bioinformatics 2006 14 617 629 10.1002/prot.20787 16320312

[B15] Vincent M Passerini A Labbe M Frasconi P A simplified approach to disulfide connectivity prediction from protein sequences BMC Bioinformatics 2008 14 20 18194539 10.1186/1471-2105-9-20 PMC2375136

[B16] Wang G Dunbrack R PISCES: a protein sequence culling server Bioinformatics 2003 14 12 1589 1591 12912846 10.1093/bioinformatics/btg224

[B17] Altschul S Madden T Schaffer A Zhang J Zhang Z Miller W Lipman D Gapped BLAST and PSI-BLAST: a new generation of protein database search programs Nucleic Acids Research 1997 14 3389 3402 9254694 10.1093/nar/25.17.3389 PMC146917

[B18] Kabsch W Sander C Dictionary of protein secondary structure: pattern recognition of hydrogen-bonded and geometrical features Biopolymers 1983 14 2577 2637 6667333 10.1002/bip.360221211

[B19] Kinch L Shi S Cheng H Cong Q Pei J Mariani V Schwede T Grishin N CASP9 target classification Proteins 2011 14 Suppl 10 21 36 21997778 10.1002/prot.23190 PMC3226894

[B20] Ahmad S Gromiha M Sarai A Real value prediction of solvent accessibility from amino acid sequence Proteins 2003 14 629 635 12577269 10.1002/prot.10328

[B21] Carugo O Predicting residue solvent accessibility from protein sequence by considering the sequence environment Protein Eng 2000 14 607 609 11054454 10.1093/protein/13.9.607

[B22] Vonderviszt F Matrai G Simon I Characteristic sequential residue environment of amino acids in proteins Int J Peptide Protein Res 1986 14 483 492 3733319 10.1111/j.1399-3011.1986.tb01046.x

[B23] Sevier C Kaiser C Formation and transfer of disulphide bonds in living cells Nature Reviews Molecular Cell Biology 2002 14 836 847 12415301 10.1038/nrm954

[B24] Washington A Singh G Diametrically opposed effects of hypoxia and oxidative stress on two viral transactivators Virology Journal 2010 14 93 20459757 10.1186/1743-422X-7-93 PMC2874542

[B25] Kim Y Otterson G Kratzke R Coxon A Kaye F Differential specificity for binding of retinoblastoma binding protein 2 to RB, p107, and TATA-binding protein Mol Cell Biol 1994 14 11 7256 7264 7935440 10.1128/mcb.14.11.7256-7264.1994 PMC359260

[B26] Jung Y Bonagura C Tilley G Gao-Sheridan H Armstrong F Stout C Burgess B Structure of C42D Azotobacter vinelandii FdI. A Cys-X-X-Asp-X-X-Cys motif ligates an air-stable [4Fe-4S]2+/+ cluster J Biol Chem 2000 14 47 36974 36983 10961993 10.1074/jbc.M004947200

[B27] Sippl M Calculation of conformational ensembles from potentials of mean force - an approach to the knowledge-based prediction of local structures in globular proteins J Mol Biol 1990 14 859 883 2359125 10.1016/s0022-2836(05)80269-4

[B28] Rata I Li Y Jakobsson E Backbone Statistical Potential from Local Sequence-Structure Interactions in Protein Loops Journal of Physical Chemistry B 2010 14 5 1859 1869 10.1021/jp909874g 20070091

